# Bibliographical Notice

**Published:** 1839-03-02

**Authors:** 


### BIBLIOGRAPHICAL NOTICE.

*An Essay on Scarlatina.* By James August Cross, M. D., Professor in Transylvania University, &c. Lexington: 1838.*

Dr. Cross has given a complete history of this disease, which of course differs but little from those already published. The most novel points of his essay are the stress which he lays upon the lesion occurring in the bronchial mucous membrane, during the course of the disease. This lesion is the congestion of the bronchial mucous membrane. Dr. Cross ascribes to this secondary condition, the functional alterations of the nervous system, which are so striking in severe, or, as they are often termed, malignant cases of scarlatina.

In these cases, he states, that the fever, from having been inflammatory, "will become decidedly typhoid, and patients, in general, will sink with great rapidity into a hopeless state of debility, under the depressing influence of an imperfectly arterialized blood. That this should result from the pathological condition of the bronchial mucous membrane, to which reference has been made, must be perfectly apparent to those the least acquainted with the physiological relations of the functions of respiration with the rest of the system." We certainly agree with Dr. Cross, that

* We received this paper some months since, and at that time, designed giving an early notice of it. From accidental causes, this was not done, and we are glad to have an opportunity of repairing this omission.

a congested state of the bronchial mucous membrane is frequent in scarlatina, but we by no means think that this congestion is the cause of the cerebral and nervous symptoms. On the contrary, we are much more disposed to regard the affection of the pulmonary organs as produced in part by the alteration of the nervous system which always takes place in severe forms of the disease, and, in part, by the general fulness, and excitement of the capillary circulation.

We have deduced conclusive proof of this fact from pathological anatomy. In the first place, we have ascertained, by strict observation, that the congestion of the bronchial mucous membrane in scarlatina is not nearly so evident as it is in measles or pneumonia, both of which diseases are much less frequently "typhoid" in their character than scarlatina. We are quite sure that a similar error is often made, in speaking with reference both to scarlatina and to measles, and that the congestion which is often observed in the bronchial mucous membrane, is by no means proportioned to the malignant character of the affection.

In the treatment, Dr. Cross relies almost entirely on emetics. These, he thinks, will be sufficient to prevent the congestion of the inflammation of the bronchial mucous membrane, or to remove it after it has occurred. Dr. Cross does not, we think, lay too much stress on emetics when he regards them as the best means of obviating the pulmonary congestion, but we must decidedly object to the almost unqualified terms in which he insists upon the necessity of frequent repetition of emetics whenever the wheezing noise should be heard in respiration, as often as three or four, or half-a-dozen times, or even more, in the twenty-four hours. The particular epidemic in which Dr. Cross found that emetics were beneficial, may not again occur with the same characters; for, in no disease do epidemics differ more completely than those of scarlatina. We fear that he may have committed an error in generalizing too widely, and in recommending a mode which is certainly not exempt from danger. If ipecacuanha only be used, the danger is not a little diminished, but it still exists, and we should always be cautious in the use of these remedies. The other remedies commonly used in the management of scarlatina, are mentioned by Dr. Cross. He estimates their value, in a spirit of impartial discussion, and very properly condemns the use of blisters and of blood-letting, when the nervous system is evidently in an irritable condition. Of these remedies, cold and warm affusion and applications to the skin, are, of course, amongst the most important.

---

*Practical Surgery.* By Robert Liston, Surgeon. *With Notes and Additional Illustrations.* By George W. Norris, M. D., *one of the Surgeons of the Pennsylvania Hospital.* Philadelphia, 1838. 8vo., pp. 374.

Liston's Practical Surgery has been for some time before the profession, both of Great Britain and our own country. The London edition was published in 1837, and was noticed in an early number of the first volume of the Examiner. It has been presented to the American reader during the current year, in the American Medical Library, with notes and additions, by Dr. Norris; and a separate edition of the same has since been issued. We have before expressed the opinion, that the work of Mr. Liston is an excellent manual of practical surgery. To the American surgeon, the annotations and additions of Dr. Norris are of much value; upon the author, they are a not unmerited reflection. Dr. Norris has annexed to his edition the improvements in operative surgery, by American surgeons, which Mr. Liston has done himself and his British readers the injustice to pass by unnoticed. The editor has also incorporated with the work, the details of several interesting cases, drawn from his experience in the Pennsylvania Hospital, which illustrate some interesting surgical points. He has, moreover, favoured us with an outline of the practice in that institution, in some important affections. This is particularly the case with the subject of fractures. These injuries are treated with marked success in Dr. Norris's hospital, and he has given the routine of the practice employed, in the cases which offer any important peculiarities.

In noticing the modification of Desault's apparatus for the treatment of fractured thighs, in use in the Pennsylvania Hospital, Dr. Norris has inadvertently given Dr. Physick credit for all the improvements there adopted. The suggestion of the block at the lower end of the outer splint, which accomplishes extension in the line of the axis of the limb, originated, we believe, with Dr. Hutchinson, a surgeon of much promise, who was cut off early in his career. He is entitled to the merit of this important improvement in manipulative surgery.

